# Integrated Bulk and Single-cell RNA Sequencing Data Constructs and Validates a Prognostic Model for Non-small Cell Lung Cancer

**DOI:** 10.7150/jca.90768

**Published:** 2024-01-01

**Authors:** Junkai Zhu, Junluo Yang, Xinyi Chen, Yang Wang, Xin Wang, Mengmeng Zhao, Guanjie Li, Yuhang Wang, Yuyao Zhu, Fangrong Yan, Tiantian Liu, Liyun Jiang

**Affiliations:** 1 Research Center of Biostatistics and Computational Pharmacy, China Pharmaceutical University, Nanjing 210009, P.R. China.; 2 Department of Radiology, Zhujiang Hospital, Southern Medical University, 253 Gongye Middle Avenue, Haizhu District, Guangzhou, Guangdong, 510282, P.R. China.

**Keywords:** non-small cell lung cancer, scRNA-seq, prognostic model, immunotherapy, drug sensitivity

## Abstract

**Background**: Most of the current research on prognostic model construction for non-small cell lung cancer (NSCLC) only involves in bulk RNA-seq data without integration of single-cell RNA-seq (scRNA-seq) data. Besides, most of the prognostic models are constructed by predictive genes, ignoring other predictive variables such as clinical features.

**Methods**: We obtained scRNA-seq data from GEO database and bulk RNA-seq data from TCGA database. We construct a prognostic model through the Least Absolute Shrinkage and Selection Operator (LASSO) and Cox regression. Furthermore, we performed ESTIMATE, CIBERSORT, immune checkpoint-related analyses and compared drug sensitivity using pRRophetic method judged by IC50 between different risk groups.

**Results**: 14 tumor-related genes were extracted for model construction. The AUC for 1-, 3-, and 5 years overall survival prediction in TCGA and three validation cohorts were almost higher than 0.65, some of which were even higher than 0.7, even 0.8. Besides, calibration curves suggested no departure between model prediction and perfect fit. Additionally, immune-related and drug sensitivity results revealed potential targets and strategies for treatment, which can provide clinical guidance.

**Conclusion**: We integrated traditional bulk RNA-seq and scRNA-seq data, along with predictive clinical features to develop a prognostic model for patients with NSCLC. According to the constructed model, patients in different groups can follow precise and individual therapeutic schedules based on immune characteristics as well as drug sensitivity.

## Introduction

Lung cancer is the leading cause of cancer-related deaths worldwide, with non-small cell lung cancer (NSCLC) comprising 85 % of lung cancers where lung adenocarcinoma (LUAD) and lung squamous cell carcinoma (LUSC) are the most common subtypes^1^, ^2^. In recent years, more and more promising treatment strategies for patients with NSCLC have been proposed and implemented, including immunotherapy and chemotherapy drugs. However, not all patients with NSCLC can benefit from those promising treatment strategies, some of which had little response to immune checkpoint inhibitors like programmed death 1 (PD1), programmed death-ligand 1 (PD-L1), etc.^3^, ^4^. Response to treatment is tightly associated with many factors such as the expression of specific genes, clinical features, immune cell infiltration, etc. Thus, it is necessary to construct a predictive model for patient stratification with considering the gene expressions and clinical features. Based on patient stratification, we can find out patients' responses to different treatment strategies and adopt appropriate ones for patients in different groups, which is in line with the principles of precise treatment and rational drug use.

Through literature review on prognostic model development for NSCLC, we found most of them focusing on immunity, metabolism, etc^5^, ^6^. However, most of the current researches only involve bulk RNA-seq data without integration of single-cell RNA-seq (scRNA-seq) data, which ignores the effect of cell heterogeneity. Moreover, most of the prognostic models are constructed by predictive genes, ignoring other predictive variables such as clinical features, including age, gender, tumor stage, etc., which might cause the inefficiency of prognostic model.

Recently, with the rapid development of sequencing technologies, scRNA-seq has been widely used as an innovative technology to investigate the transcriptome of different cell types^7^. Although scRNA-seq is relatively expensive, the information from it can be quite meaningful. Compared with the traditional bulk RNA-seq which mainly concentrates on the average expression of all cells in one patient, scRNA-seq can detect cellular and molecular changes in tumor cells^7^. Additionally, as scRNA-seq highlights intratumor heterogeneity and distinct subpopulations, we can quantify heterogeneous make-up of immune cells infiltration in normal and tumor tissues^8^, ^9^, which is a key factor for treatment response and prognosis in NSCLC^10^, ^11^.

In summary, this study aims to construct a prognostic model by integrating scRNA-seq, bulk RNA-seq and other clinical features to stratify patients with NSCLC based on the risk score. Besides, we also performed analyses on scRNA-seq and bulk RNA-seq data respectively, as well as analyses between high-risk and low-risk groups such as immune-related analysis, drug sensitivity analysis, etc.

## Materials and Methods

### Data acquisition and processing

All datasets used for analyses in this article were acquired from public databases. The information of these datasets was shown in detail (Table 1). First, a 10x scRNA-seq dataset (GSE117570) containing four NSCLC tumor samples as well as four normal samples were downloaded from Gene Expression Omnibus (GEO) database. After combination and quality control (QC), a dataset with 3146 features and 9882 cells for prognostic model development and analyses was obtained. Besides, we acquired bulk RNA-seq data as well as clinical data from The Cancer Genome Atlas (TCGA) database. We converted the “fpkm” values to “tpm” values for bulk RNA-seq data, which were performed log_2_ transformation after adding 1. As for clinical data, the information of survival time, survival status, age, gender, tumor stage and so on was extracted. Furthermore, after merging the processed bulk RNA-seq data with processed clinical data, a dataset with 966 patients was finally obtained for prognostic model construction and analyses.

In order to validate model prediction performance and other findings, 3 validation datasets, including 172 NSCLC patients (GSE42127), 117 LUAD patients (GSE13213) and 248 LUSC patients (GSE157009) were acquired from GEO database. We performed log_2_ transformation after adding 1 for bulk RNA-seq data and then normalized the data, which was finally integrated with clinical data containing information of survival time, survival status, age, gender, tumor stage, etc.

### Processing and analysis on scRNA-seq data

“Seurat” R package^12^ was adopted to process and analyze the scRNA-seq data. First, we integrated the tumor and normal samples, then converted the combined data into a Seurat object. After that, we performed QC to extract the genes which expressed in more than 3 cells as well as the cells with more than 500 but less than 2000 expression signatures. Furthermore, we filtered the mitochondrial and ribosomal genes which expressed in more than 20% cells. Next, we normalized the filtered data through “LogNormalize” method and found the top 1500 highly variable genes based on the variance stabilization transformation (vst). At the same time, we scaled and run principal component analysis (PCA) on the normalized data with selected 1500 highly variable genes. Finally, we determined the cluster number by “JackStraw” and “ScoreJackStraw” functions, then clustered the cells using “FindNeighbors” and “FindClusters” functions. To visualize the clustering results, uniform manifold approximation and projection (UMAP) algorithm was adopted for data dimensionality reduction. In addition, according to the clustering results, we used “SingleR” R package^13^ to automatically annotate cell types, and “Monocle” R package^14^ to perform cell trajectory and pseudo-time analysis. We also run gene set enrichment analysis (GSEA) as well as gene set variation analysis (GSVA) through “clusterProfiler” R package^15^ and “GSVA” R package^16^ separately. Most importantly, to identify the tumor-related clusters, we calculated the proportions of tumor cells and normal cells in each cluster. Clusters with proportion ratios (proportions of tumor cells / proportions of normal cells) that were higher than the threshold of 2 would be regarded as tumor-related clusters. The markers from tumor-related clusters derived from “FindAllMarkers” function were chosen for prognostic model development.

### Processing and analysis on bulk RNA-seq data

First, we performed differential expression analysis (DEA) between tumor samples and normal samples by “limma” R package^17^, using |log_e_FC|>0.5 and false discovery rate (FDR) < 0.05 as cut-off value. Furthermore, Gene Ontology (GO) and Kyoto Encyclopedia of Genes and Genomes (KEGG) enrichment analyses were utilized to investigate the most significantly enriched pathways and biological processes based on the differential expression analysis results through “clusterProfiler” package. Subsequently, Weighted Gene Correlation Network Analysis (WGCNA) was exploited to cluster the genes into different modules and find out the module with the highest correlation to tumor phenotype using “WGCNA” R package^18^. The genes in that module were also selected for prognostic model development.

### Development and validation of prognostic model using common genes from both scRNA-seq data and bulk RNA-seq data

We first generated a Venn diagram using “VennDiagram” R package to find out common genes from both scRNA-seq data and bulk RNA-seq data and then merged clinical data with bulk RNA expression data containing common genes. Then, Least Absolute Shrinkage and Selection Operator (LASSO) Cox regression analysis was performed to select genes with nonzero coefficients through “glmnet” R package^19^. Subsequently, we further extracted genes to construct a gene score via multivariate Cox regression analysis by “survival” R package. The formula for the gene score _i_s listed below:


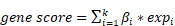

(1)

In the formula, 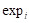 represents the expression value of the *i*^th^ chosen gene and 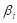 refers to corresponding coefficient in multivariate Cox regression analysis. Along with the gene score, we performed univariate as well as multivariate Cox regression analyses on clinical features, including age, gender and tumor stage. The variables significantly correlated with prognosis were selected for model development. After the model construction, we obtained the coefficients of selected variables by multivariate Cox regression analysis and utilized the model to calculate risk score for each patient. According to the median of risk scores, we divided patients into high-risk group (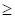
median) and low-risk group (<median). At the same time, we calculated risk scores for three validation cohorts respectively based on the constructed model and divided the patients into high-risk group and low-risk group based on the median of risk scores separately.

For validation, survival curves were drawn to visualize survival difference between high and low risk groups by “survival” and “survminer” R packages. In addition, we also generated risk plots to investigate the relationship between risk group and survival status. To test the discrimination, we utilized "timeROC" R package^20^ to generate receiver operating characteristic (ROC) curves and calculated the area under the curves (AUC) for 1-, 3-, and 5 years to evaluate the model performance in predicting overall survival (OS). To test the calibration, we also used “rms” R package to draw 1-, 3-, and 5 years of calibration curves. For the convenience of application, we converted the model into a nomogram through “rms” R package, which can be easily utilized to predict 1-, 3-, and 5 years OS by doctors, even by patients.

### Association between risk score and gene score as well as clinical features

We generated box-violin plots to investigate the association between risk score and gene score, as well as the relationship between risk score and clinical features containing age, gender and tumor stage in TCGA cohort through R package “ggpubr”.

### Gene set enrichment analysis between high-risk and low-risk groups

We performed gene set enrichment analysis (GSEA) in TCGA cohort to find out and visualize the most significantly enriched GO and KEGG pathways between high-risk and low-risk groups using R packages “org.Hs.eg.db”, “clusterProfiler” and “enrichplot”.

### Immune-related analyses between high-risk and low-risk groups

In TCGA cohort, we first investigated the relationship between risk groups and infiltration levels of various types of immune cells by calculating ESTIMATE Score, Immune Score, Stromal Score and Tumor Purity through R package “estimate”^21^ to estimate the effect of prognostic model on the tumor immune microenvironment (TIME) of NSCLC. Then, we generated box-violin plots to visualize comparison results. Besides, we performed CIBERSORT analysis^22^ to compare relative proportions of various immune cells in tissues of high-risk and low-risk groups. We also compared expression of immune checkpoint related genes. Box plots were drawn to visualize comparison results.

### Drug sensitivity analysis between high-risk and low-risk groups

R package “pRRophetic”^23^ used baseline gene expression and *in vitro* drug sensitivity derived from cell lines, coupled with *in vivo* baseline tumor gene expression to predict patients' response to drugs. To choose proper drugs for patients in different risk groups, we utilized R package “pRRophetic” to perform drug sensitivity analysis between high-risk and low-risk groups judged by IC_50_ in TCGA cohort, and then generated boxplots and correlation diagrams for comparison visualization.

### Statistical analyses and visualization

R software (version 4.1.1) on CentOS was used for statistical analyses. Proportion test was used to compare the proportion difference of tumor and normal cells. The non-parameter Wilcoxon rank-sum test was performed to test difference on continuous variables between two groups, while Kruskal-Wallis test for three and three more groups. Additionally, we performed LASSO regression and Cox regression analyses to develop prognostic model, as well as Kaplan-Meier survival analysis to test survival difference between high-risk and low-risk groups using log-rank test. A two-sided p-value < 0.05 indicated statistical significance. For results visualization, we used R packages “ggplot2” and “ggpubr”.

## Results

### Identification of markers for tumor-related clusters using scRNA-seq data

**Figure 1:**
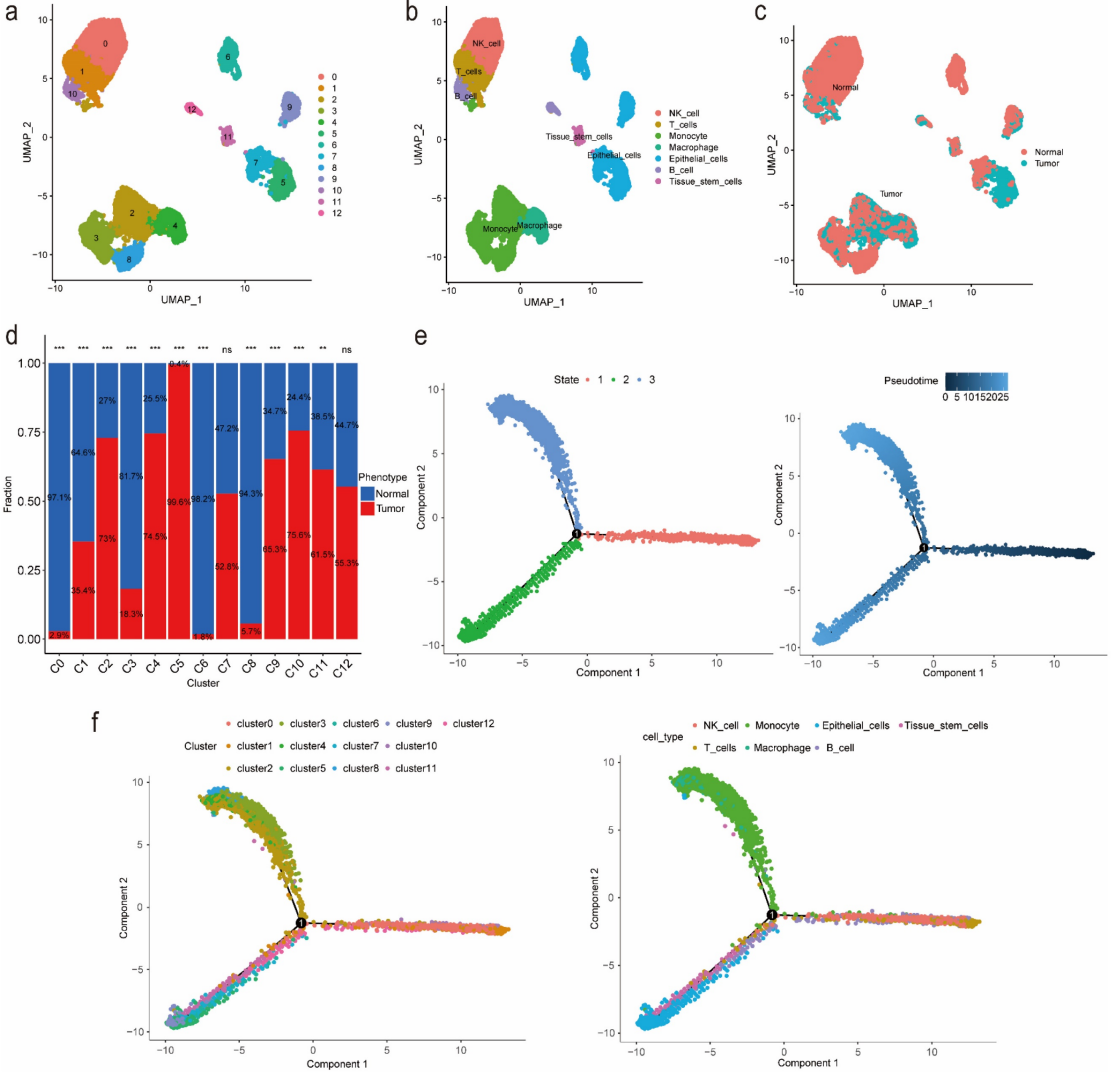
Identification of markers for tumor-related clusters using scRNA-seq data. (a) Identification and visualization of 13 clusters. (b) Annotation and visualization of 7 cell types. (c) Distribution of tumor cells and normal cells in each cluster. (d) The proportions of tumor cells and normal cells in each cluster. (e-f) Cell trajectory and pseudo-time analysis for the identified clusters and cell types. Abbreviations: scRNA-seq, single-cell RNA-seq. Symbols: ***, 0 < p-value < 0.001; **, 0.001 ≤ p-value < 0.01; *, 0.01 ≤ p-value < 0.05; ns, 0.05 ≤ p-value < 1.

We identified 13 clusters and further automatically annotated 7 cell types, including NK cell covered cluster 0, T cells covered cluster 1, Monocyte covered cluster 2, cluster 3 and cluster 8, Macrophage covered cluster 4, Epithelial cells covered cluster 5, cluster 6, cluster 7 and cluster 9, B cell covered cluster 10, cluster 12 and Tissue stem cells covered cluster 11 (Figure 1a, 1b). Besides, we also focused on the phenotype and found that tumor cells mainly distributed in cluster 2, cluster 4, cluster 5 and cluster 10, while normal cells mainly distributed in cluster 0, cluster 3, cluster 6 and cluster 8 (Figure 1c, 1d; Table S1). Then we performed gene set variation analysis (GSVA) and the results suggested that in up-regulated pathways, cluster 4 and cluster 5, Macrophage and Epithelial cells, were enriched in the cellular component related pathways such as endoplasmic reticulum as well as involved in the process of immune effector, cell activation and organophosphate biosynthetic, while in down-regulated pathways, cluster 4 and Macrophage were enriched in Ribosome composition-related pathways and involved in the process of protein localization to the endoplasmic reticulum, peptide metabolism, viral gene expression, translational initiation and cotranslational protein targeting to membrane (Figure S1, S2). Additionally, according to the cell trajectory and pseudo-time analysis results, Monocyte and Macrophage, which covered cluster 2 and cluster 4 respectively, only corresponded to state 3, while Epithelial cells covered cluster 5 only corresponded to state 2. Both state 2 and state 3 were in later stages of cell trajectory development (Figure 1e, 1f). Finally, we regarded cluster 2, cluster 4, cluster 5 and cluster 10 as tumor-related clusters and extracted the markers for prognostic model development.

### Identification of tumor-related module genes using bulk RNA-seq data

**Figure 2:**
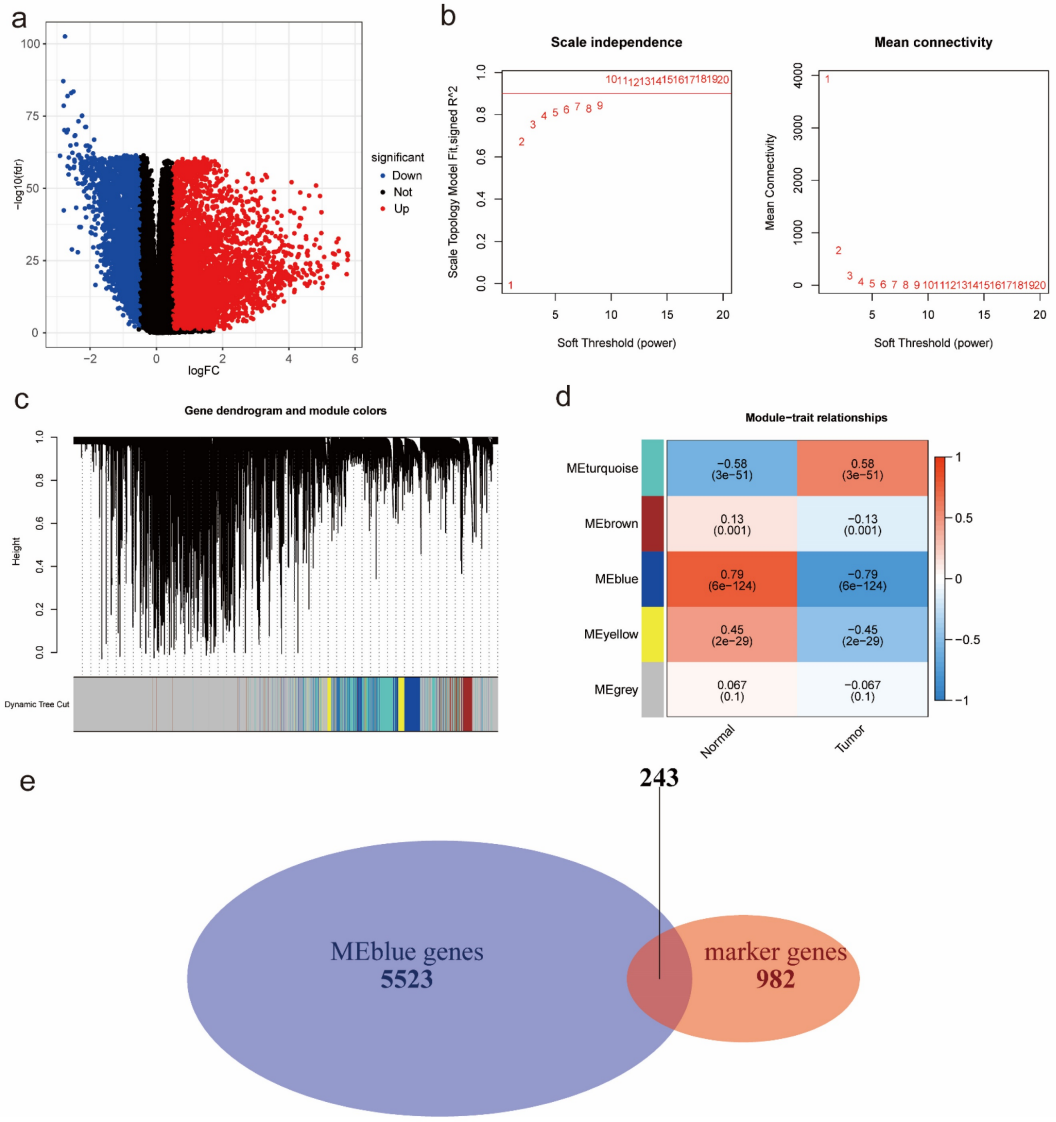
Identification of tumor-related module genes using bulk RNA-seq data. (a) A volcano plot to visualize up-regulated and down-regulated genes based on DEA results. (b) The scale-free fit index for soft thresholding powers. The soft thresholding power *β* in the WGCNA was determined based on a scale-free R^2^ (R^2^=0.9). The left panel illustrates the relationship between *β* and R^2^. The right panel illustrates the relationship between *β* and mean connectivity. (c) A dendrogram of the DEGs clustered based on different metrics. (d) A heatmap indicates the correlation between gene modules and phenotypes (normal & tumor). (e) A Venn diagram to extract 243 common genes between markers from tumor-related clusters in scRNA-seq data and genes from tumor-related “blue” module in bulk RNA-seq data. Abbreviations: DEA, differential expression analysis; DEGs, differential expression genes; WGCNA, Weighted Gene Correlation Network Analysis; scRNA-seq, single-cell RNA-seq.

According to the DEA results, there were 5942 up-regulated genes and 3172 down-regulated genes. We generated a volcano plot to visualize the distribution of the differential expression genes (DEGs) (Figure 2a). Moreover, the results of GO enrichment analysis indicated that DEGs were mainly enriched in ion transmembrane transport regulation, channel and signaling receptor activity, as well as the component of apical part of cell, synaptic membrane, apical plasma membrane and transporter complex (Figure S3a). The results of KEGG enrichment analysis revealed that DEGs were mainly involved in Neuroactive ligand-receptor interaction and Cytokine-cytokine receptor interaction, Calcium signaling pathway and cAMP signaling pathway, Alcoholism and Neutrophil extracellular trap formation (Figure S3b). Finally, we performed WGCNA analysis to identify tumor-related module genes. We observed that the soft thresholding power 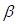 was 10 when the fit index of scale-free topology reached 0.9 in the process of co-expression network construction (Figure 2b). Based on the soft thresholding power as well as the average linkage hierarchical clustering, we identified 5 gene modules (Figure 2c). The phenotypic correlation analysis showed that the “blue” module was most significantly correlated with tumor phenotype according to the correlation coefficient and p-value (Figure 2d). Combined with 1225 markers from tumor-related clusters in scRNA-seq data and 5766 genes from “blue” module in bulk RNA-seq data, 243 common genes were extracted for prognostic model construction (Figure 2e).

### Prognostic model development and validation using common genes from both scRNA-seq data and bulk RNA-seq data

**Figure 3:**
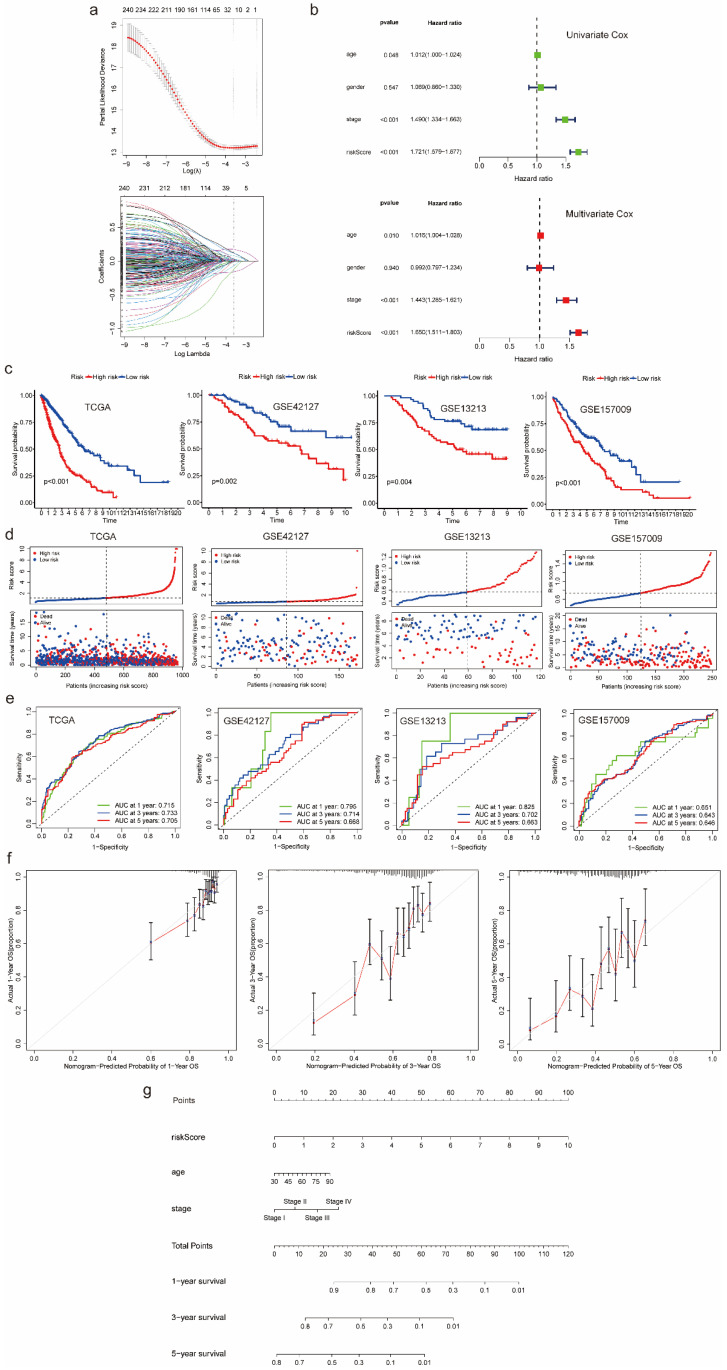
Prognostic model development and validation using common genes from both scRNA-seq and bulk RNA-seq data. (a) 20 genes with nonzero coefficients were chosen for multivariate Cox regression analysis through LASSO Cox regression analysis. (b) Univariate and multivariate Cox regression analyses to select predictive clinical features with independent prognostic ability as well as test the independent prognostic ability of risk score. (c) Survival curves to evaluate the patient stratification ability of the constructed prognostic model in the TCGA, GSE42127, GSE13213 and GSE157009 cohorts. (d) Risk score distribution and patient status for TCGA, GSE42127, GSE13213 and GSE157009 cohorts. (e) ROC curves to evaluate the OS prediction performance of the constructed prognostic model in the TCGA, GSE42127, GSE13213 and GSE157009 cohorts. (f) Calibration curves to test departure between model prediction and perfect fit in the TCGA cohort. (g) A nomogram converted from the constructed prognostic model. Abbreviations: scRNA-seq, single-cell RNA-seq; LASSO, Least Absolute Shrinkage and Selection Operator; TCGA, The Cancer Genome Atlas; ROC, receiver operating characteristic; OS, overall survival.

According to the results of LASSO Cox regression analysis, 20 potential prognostic genes were identified from common genes (Figure 3a). We further extracted 14 prognostic genes via multivariate Cox regression analysis and then constructed a gene score:

*gene score* = 0.178 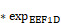 + 0.163 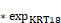 + 0.261 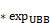 + 0.268 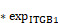 + 0.097 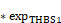 - 0.089 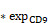 - 0.182 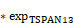 - 0.357 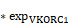 - 0.218 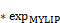 + 0.281 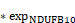 + 0.149 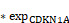 - 0.423 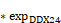 + 0.220 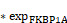 + 0.210 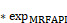

(2)

Then we identified variables “gene score”, “age” as well as “stage” which were significantly correlated with prognosis in univariate and multivariate Cox regression analyses at the same time (Figure 3b). After that, we utilized selected variables to develop a prognostic model and calculate risk score which was used to divide patients into high-risk and low-risk groups:

*risk score* = 0.501 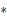 *gene score* + 0.016 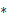 *age* + 0.355 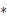 *stage2* + 0.735 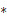 *stage3* + 1.096 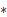 *stage4* (3)

Comparing with the significant survival difference between high and low risk groups in TCGA cohort as well as three validation cohorts, we found patients in low-risk group had better prognosis (Figure 3c). Besides, we also observed that patients in high-risk group were associated with dead survival status, while patients in low-risk group were associated with alive survival status (Figure 3d). Additionally, as for discrimination, the prognostic model showed good performance in predicting OS of each patient. The AUC for 1-, 3-, and 5 years OS prediction in TCGA cohort and three validation cohorts were almost higher than 0.65, some of which were even higher than 0.7, even 0.8 (Figure 3e). As for calibration, the curves of 1-, 3-, and 5 years suggested no departure between model prediction and perfect fit (Figure 3f). For better application, we converted the model into a nomogram which can be conveniently used to predict 1-, 3-, and 5 years OS by doctors, even by patients (Figure 3g). Moreover, we generated a heatmap and a bubble plot to visualize the expression of model genes in each cluster identified from scRNA-Seq data (Figure S4, S5).

### Features relevance, gene set enrichment analysis between high-risk and low-risk groups

**Figure 4:**
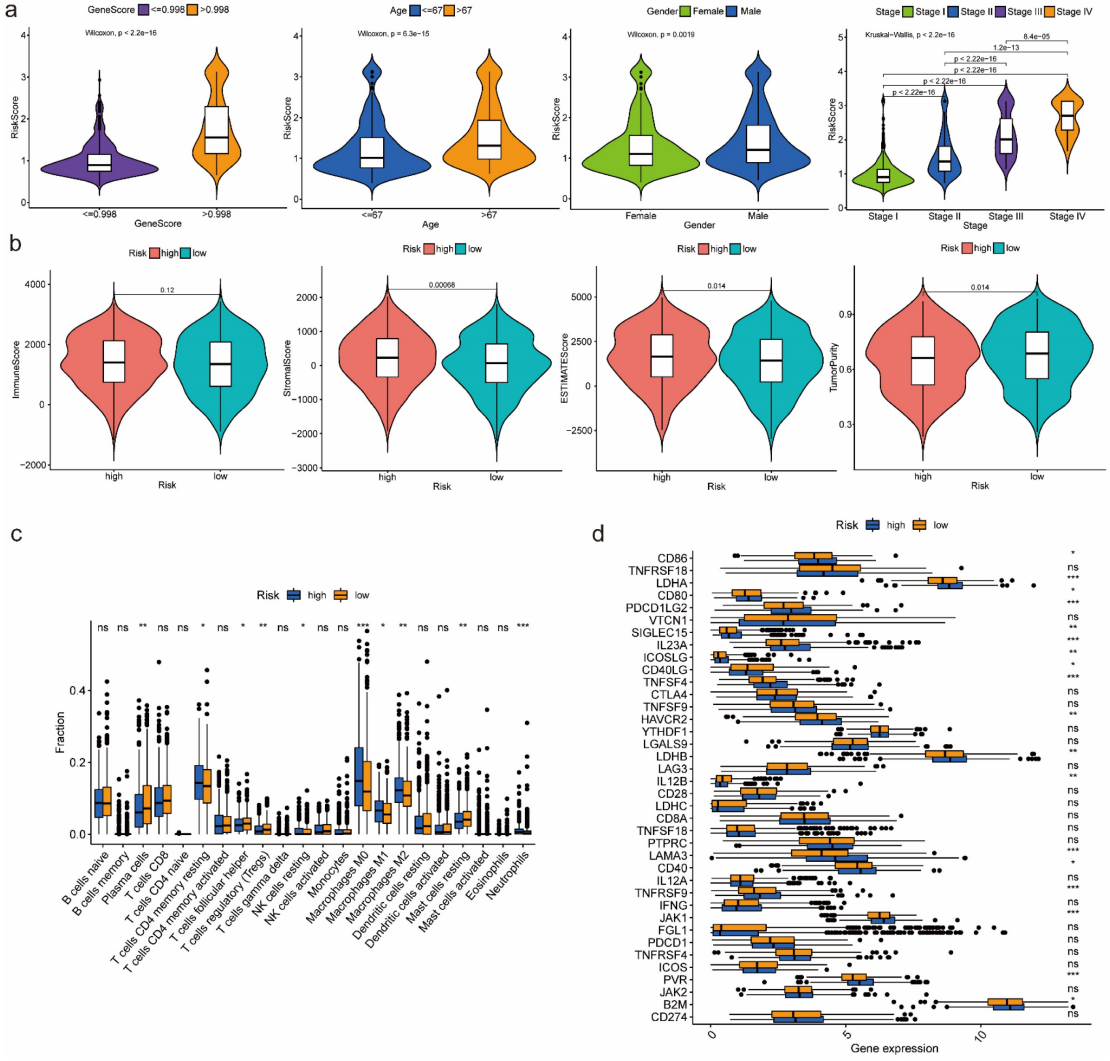
Features relevance analysis, GSEA as well as immune-related analyses between high-risk and low-risk groups. (a) The association between risk score and features including gene score, age, gender as well as tumor stage. (b) Estimation comparison of infiltration levels of various types of immune cells in TIME between high-risk and low-risk groups. (c) Relative fractions comparison of 22 immune cells in tissues between high-risk and low-risk groups. (d) Expression comparison of 38 immune checkpoint related genes between high-risk and low-risk groups. Abbreviations: GSEA, gene set enrichment analysis; TIME, tumor immune microenvironment. Symbols: ***, 0 < p-value < 0.001; **, 0.001 ≤ p-value < 0.01; *, 0.01 ≤ p-value < 0.05; ns, 0.05 ≤ p-value < 1.

According to the box-violin plots, we found risk score was significantly associated with gene score as well as clinical features including age, gender and tumor stage. Specifically, we divided patients into two groups based on the median of gene score and the median of age respectively. We observed that older patients and patients with higher gene scores had higher risk scores. We also noticed that male patients and patients in Stage IV had higher risk scores (Figure 4a).

For GO pathways, GSEA analysis indicated that genes in high-risk group most significantly enriched in molecular function of ferric iron binding and biological process of microtubule cytoskeleton organization involved in mitosis, nuclear transcribed mRNA catabolic process nonsense mediated decay, regulation of CGMP mediated signaling and response to ionizing radiation. However, genes in low-risk group most significantly enriched in biological process of coronary vasculature morphogenesis, G protein coupled receptor signaling pathway involved in heart process, regulation of cardiac muscle contraction by calcium ion signaling, regulation of cardiac muscle contraction by regulation of the release of sequestered calcium ion and regulation of metallopeptidase activity (Figure S6, S7).

For KEGG pathways, we found that genes in high-risk group most significantly enriched in cell cycle, DNA replication, ECM receptor interaction, focal adhesion and P53 signaling related pathways, while genes in low-risk group most significantly enriched in cytosolic DNA sensing, olfactory transduction and regulation of autophagy related pathways (Figure S8).

### Immune-related analyses results between high-risk and low-risk groups

Estimation of infiltration levels of various types of immune cells in TIME revealed that there was no significant difference on immune score between high-risk and low-risk groups. Stromal score and ESTIMATE score were higher in high-risk group, while tumor purity was higher in low-risk group (Figure 4b). CIBERSORT analysis results indicated that the relative fractions of T cells CD4 memory resting, NK cells resting, Macrophages M0, Macrophages M1, Macrophages M2 and Neutrophils in tissues were higher in high-risk group, while the relative fractions of Plasma cells, T cells follicular helper, T cells regulatory and Mast cells resting were higher in low-risk group (Figure 4c). Comparing the expression of 38 immune checkpoint related genes, we found that high-risk group was significantly associated with up-regulation of CD86, LDHA, CD80, PDCD1LG2, SIGLEC15, IL23A, ICOSLG, TNFSF4, HAVCR2, LDHB, LAMA3, CD40, TNFRSF9, JAK1, PVR and B2M, while low-risk group was significantly correlated with up-regulation of CD40LG and IL12B (Figure 4d).

### Drug sensitivity comparison between high-risk and low-risk groups

**Figure 5:**
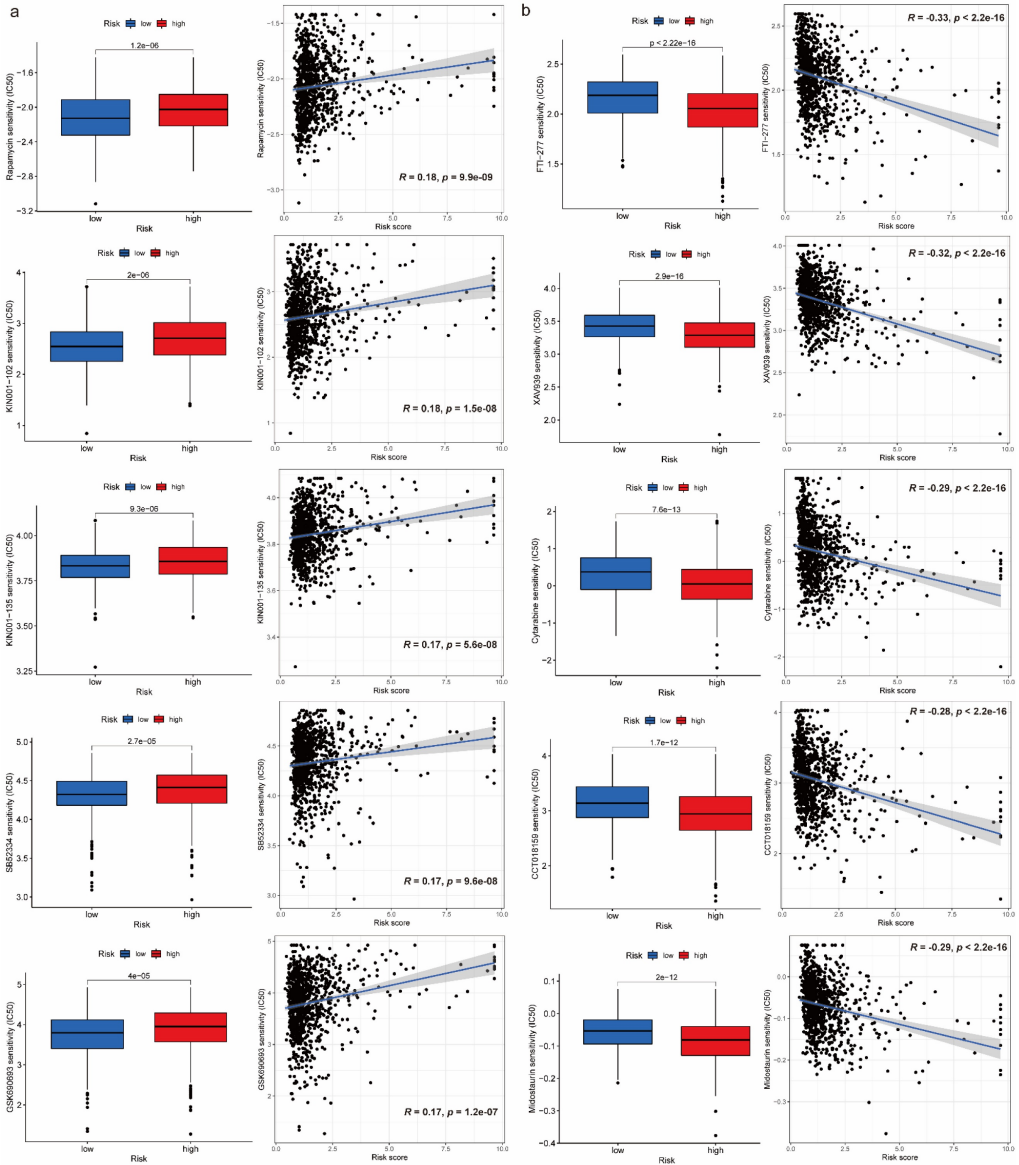
Drug sensitivity comparison between high-risk and low-risk groups. (a) Boxplots for IC50 comparison and correlation diagrams for relationship investigation between risk score and IC50 on 5 selected drugs in low-risk group. (b) Boxplots for IC50 comparison and correlation diagrams for relationship investigation between risk score and IC50 on 5 selected drugs in high-risk group.

We obtained 102 drugs which have significant difference on IC50 between high-risk and low-risk groups from GDSC 2016 drug dataset. Lower IC50 indicates better response to drug. There were 8 drugs with lower IC50 in low-risk group and 94 drugs with lower IC50 in high-risk group. Furthermore, we extracted 5 drugs including Rapamycin, KIN001-102, KIN001-135, SB52334, GSK690693 for low-risk group and FTI-277, XAV939, Cytarabine, CCT018159, Midostaurin for high-risk group respectively based on IC50 difference p-value, correlation value as well as correlation p-value (Figure 5a, 5b). Additionally, details on drug sensitivity comparison were shown in supplementary material (Table S2).

## Discussions

In this research, we developed a prognostic model for patients with NSCLC integrating scRNA-seq, bulk RNA-seq data and other predictive clinical features. As for patient stratification performance, we noticed the constructed model could effectively stratify patients in TCGA cohort into high-risk and low-risk groups, which had significant survival difference. Additionally, three external validation cohorts (NSCLC, LUAD, LUSC) were utilized to verify stratification performance, with consistent results we observed. As for OS prediction performance, the average AUC of 1-, 3-, and 5 years OS for TCGA and three validation cohorts was 0.72, 0.73, 0.73 and 0.65 separately. Comparing with the current prognostic models for NSCLC, our model has better performance on patient stratification as well as OS prediction^24^-^26^. However, prediction performance on 5 years OS and patients with LUSC needs improvement, which might be caused by insufficient samples for model construction.

Considering the composition of prognostic model, it is reported that EEF1D overexpression promotes osteosarcoma cell proliferation by facilitating Akt-mTOR and Akt-bad signaling^27^. KRT18 has been suggested to be overexpressed in most types of human tumor, which is correlated with the malignant status and acts as an oncogene in colorectal cancer^28^. High expression of UBB and ITGB1 has been demonstrated to predict worse prognosis among non-smoking patients with LUAD through bioinformatics analysis^29^. Studies have indicated the functions of THBS1 in the development of several cancers, including breast cancer, melanoma, gastric cancer, cervical cancer and glioblastoma^30^. NDUFB10, which is associated with NADH oxidation, was observed overexpressed in LIHC and LUAD tumor tissues in previous research^31^. CDKN1A functions as an oncogene, promoting cancer cell proliferation by inhibiting apoptosis in NSCLC^32^. High FKBP1A expression is correlated with a poor survival rate in LIHC patients based on the current research^33^, while the relationship between expression level of MRFAP1 and prognostic significance is unclear. On the contrary, an increased CD9 expression was associated with favorable survival in cancer patients, suggesting that CD9 expression could be a valuable survival factor in cancer patients^34^. TSPAN13 has been shown to be a tumor suppressor gene in breast cancer^35^. MYLIP was proved to have a significant inhibitory effect on the proliferation, migration, and invasion of lung cancer cells, suggesting that MYLIP may be a tumor suppressor gene for lung cancer^36^. Investigation revealed that DDX24 significantly inhibited growth of multiple cancer cell lines without affecting normal cell growth and survival, underlining its value as a drug target^37^. However, there is no enough evidence to confirm the association between expression level of VKORC1 and prognostic significance. Except the predictive genes, we also innovatively added easily available predictive clinical features to the model, which was demonstrated to improve the prediction performance. It is reported that age and tumor stage are major prognostic factors affecting survival in patients with lung cancer. The rate of mortality was higher in elderly patients, and the median survival time of elderly patients was significantly lower compared with that of younger patients based on univariate and multivariate analyses^38^. Besides, current 5-year survival estimates in NSCLC range from 73 % in stage IA disease to 13 % in stage IV disease^39^.

Additionally, immune-related analyses between high-risk and low-risk groups indicated that the constructed prognostic model was tightly associated with TIME composition and regulation, which might affect the immune response. According to the ESTIMATE Score, we found tumor purity was lower in high-risk group. Besides, there were totally 6 immune cells with higher abundance in high-risk group, while the relative fractions of 4 immune cells were higher in low-risk group based on CIBERSORT analysis results. It is reported that T cells regulatory which was higher in low-risk group plays an important role in suppressing immune responses of other cells, which may also suppress immune response to cancer cells^40^. Thus, it may be a potential target for immunotherapy to patients in low-risk group. Moreover, with the development of immunogenomics, more and more immune-related genes have been found as treatment targets. By comparing expression of 38 immune checkpoint related genes between high-risk and low-risk groups, we found 18 immune checkpoint related genes had higher expression in high-risk group, while only 2 genes had higher expression in low-risk group. Concretely, genes of CD86, LDHA, CD80, PDCD1LG2, SIGLEC15, JAK1 and B2M have been demonstrated to have negative effect on immunotherapy, while genes of IL23A, ICOSLG, TNFSF4, CD40, TNFRSF9, CD40LG and IL12B have positive effect. Therefore, we can choose different kinds of immunotherapies based on the expression of immune-related genes in different risk groups.

Like immune-related analyses, drug sensitivity comparison between high-risk and low-risk groups can help us choose proper drugs with best response to patients. It is reported that Rapamycin inhibits the growth and metastatic progression of NSCLC^41^. Besides, a strong correlation between risk scores and anticancer medication sensitivity of KIN001-102 and KIN001-135 was found in previous study^42^. PAFAH1B3 is elevated in human pan-cancer, which is correlated with greater pathology and poor prognosis, in particular for NSCLC and liver hepatocellular carcinoma (LIHC)^43^. Current research found that the expression of PAFAH1B3 was negatively correlated with drug sensitivity of SB52334^43^. Moreover, it is suggested that the combination of temsirolimus and GSK690693 could be a novel strategy for lung cancer therapy^44^. The results of current studies suggested that FTase inhibition by FTI-277 may be an effective strategy for targeting H-Ras-mediated proliferation, migration and invasion of breast cells^45^. However, there is no evidence to prove curative effects on FTI-277 against NSCLC, which may be a promising drug needs further investigation in the future. Several recent studies have demonstrated that XAV939 is able to inhibit the growth of breast, colon and non-small cell lung cancer cells by blocking the Wnt signaling pathway^46^. A case report showed that Gemcitabine is an effective drug against NSCLC and has a structure similar to cytarabine, which has been widely used in intrathecal chemotherapy^47^. According to previous study, elevated expression of nuclear HSP90 could be detected in breast cancer and NSCLC, and CCT018159 manifested the inhibitory activity of HSP90^48^. A targeted drug screen revealed that the recently approved multi-kinase inhibitor Midostaurin has potent activity in several lung cancer cells independent of its intended target, PKC, or a specific genomic marker^49^.

Nevertheless, there are also some limitations that should be improved in the future. First, all the results in this research were obtained from bioinformatic analyses, which needs to be validated through experiment. Besides, it is promising to add more predictive variables to the prognostic model, which can further improve the prediction performance on OS, and find out more valuable information.

In conclusion, we integrated traditional bulk RNA-seq and scRNA-seq data, along with predictive clinical features to develop a prognostic model for patients with NSCLC. Through verification, the model has been demonstrated to perform well enough in TCGA training cohort as well as different GEO validation cohorts. With the help of the constructed model, we can divide the patients into high-risk and low-risk groups. Patients in different groups can follow precise and individual therapeutic schedules based on immune characteristics as well as drug sensitivity comparison. Furthermore, we have converted the model to a nomogram, which can be conveniently utilized by doctors, even the patients.

## Supplementary Material

Supplementary figures and tables.

## Figures and Tables

**Table 1:** Datasets for model construction and validation

Dataset	Source	Type	Application	Cancer Type	Sample Count
GSE117570	GEO	scRNA	training	LUAD+LUSC	8
TCGA Cohort	TCGA	bulk RNA	training	LUAD+LUSC	966 (LUAD: 482; LUSC: 484)
GSE42127	GEO	bulk RNA	validation	LUAD+LUSC	172 (LUAD: 130; LUSC: 42)
GSE13213	GEO	bulk RNA	validation	LUAD	117
GSE157009	GEO	bulk RNA	validation	LUSC	248

* Abbreviations: GEO, Gene Expression Omnibus; TCGA, The Cancer Genome Atlas; scRNA, single-cell RNA; LUAD, lung adenocarcinoma; LUSC, lung squamous cell carcinoma.

## Abbreviations

NSCLC: non-small cell lung cancer
LUAD: lung adenocarcinoma
LUSC: lung squamous cell carcinoma
LIHC: liver hepatocellular carcinoma
PD1: programmed death 1
PD-L1: programmed death-ligand 1
scRNA-seq: single-cell RNA-seq
GEO: Gene Expression Omnibus
QC: quality control
TCGA: The Cancer Genome Atlas
vst: variance stabilization transformation
PCA: principal component analysis
UMAP: uniform manifold approximation and projection
DEA: differential expression analysis
FDR: false discovery rate
GO: Gene Ontology
KEGG: Kyoto Encyclopedia of Genes and Genomes
WGCNA: Weighted Gene Correlation Network Analysis
LASSO: Least Absolute Shrinkage and Selection Operator
ROC: receiver operating characteristic
AUC: the area under the curves
OS: overall survival
GSEA: gene set enrichment analysis
GSVA: gene set variation analysis
TIME: tumor immune microenvironment
DEGs: differential expression genes
