# Supplementary material for: Integrated Bulk and Single-cell RNA Sequencing Data Constructs and Validates a Prognostic Model for Non-small Cell Lung Cancer
Source: J Cancer. 2024 Jan 1;15(3):796–808. doi: 10.7150/jca.90768 (PMC10777029; doi:10.7150/jca.90768)
Supplement: Supplementary file 1 — Supplementary figures and tables. [file jcav15p0796s1.pdf]

Integrated Bulk And Single-cell RNA Sequencing Data Constructs And Validates A Prognostic Model For Non-small Cell Lung Cancer

Supplementary material

Figures 2
Tables 9

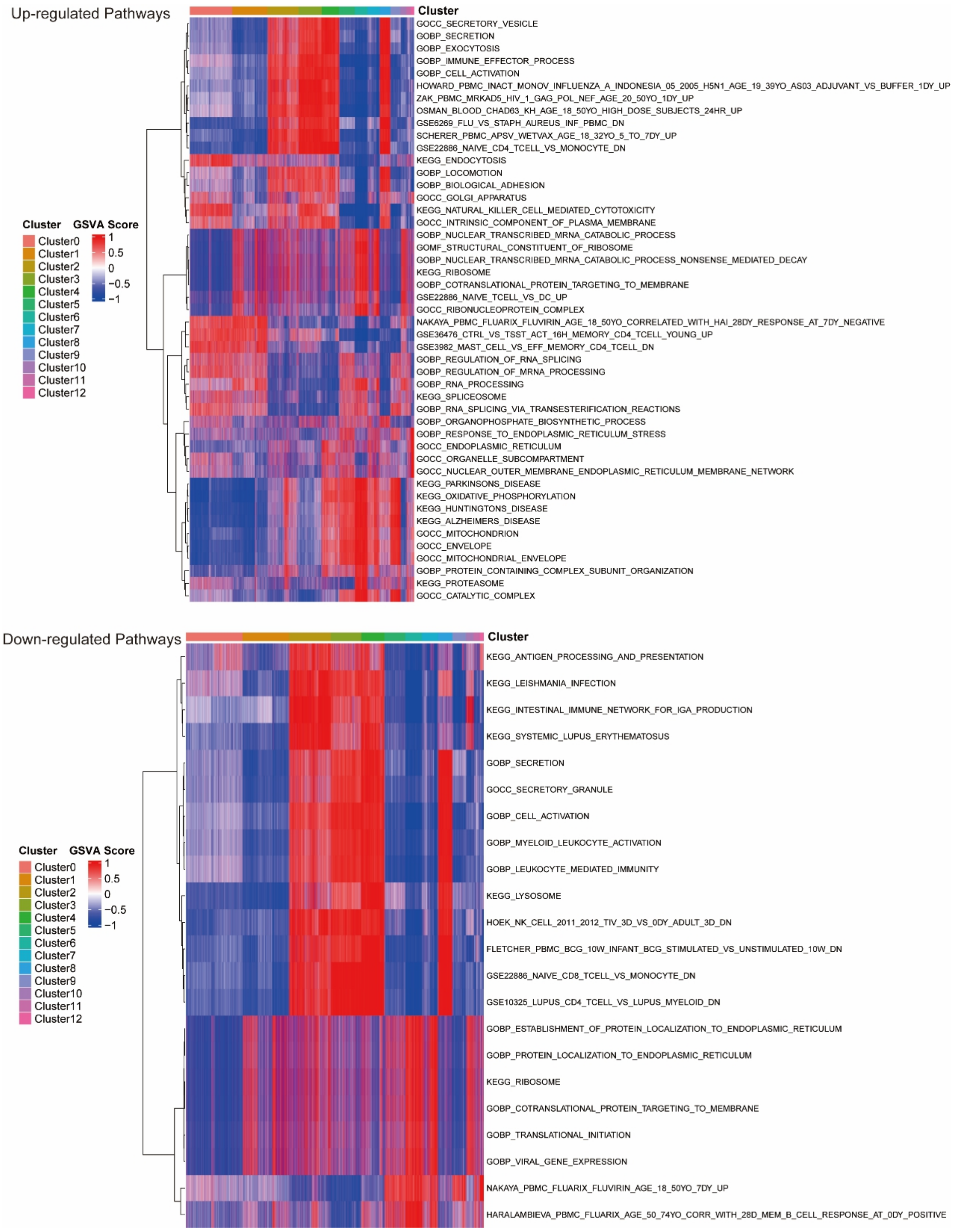

Figure S1. GSVA scores for up-regulated and down-regulated pathways in each cluster identified from scRNA-seq data. Abbreviations: GSVA, gene set variation analysis; scRNA-seq, single-cell RNA-seq

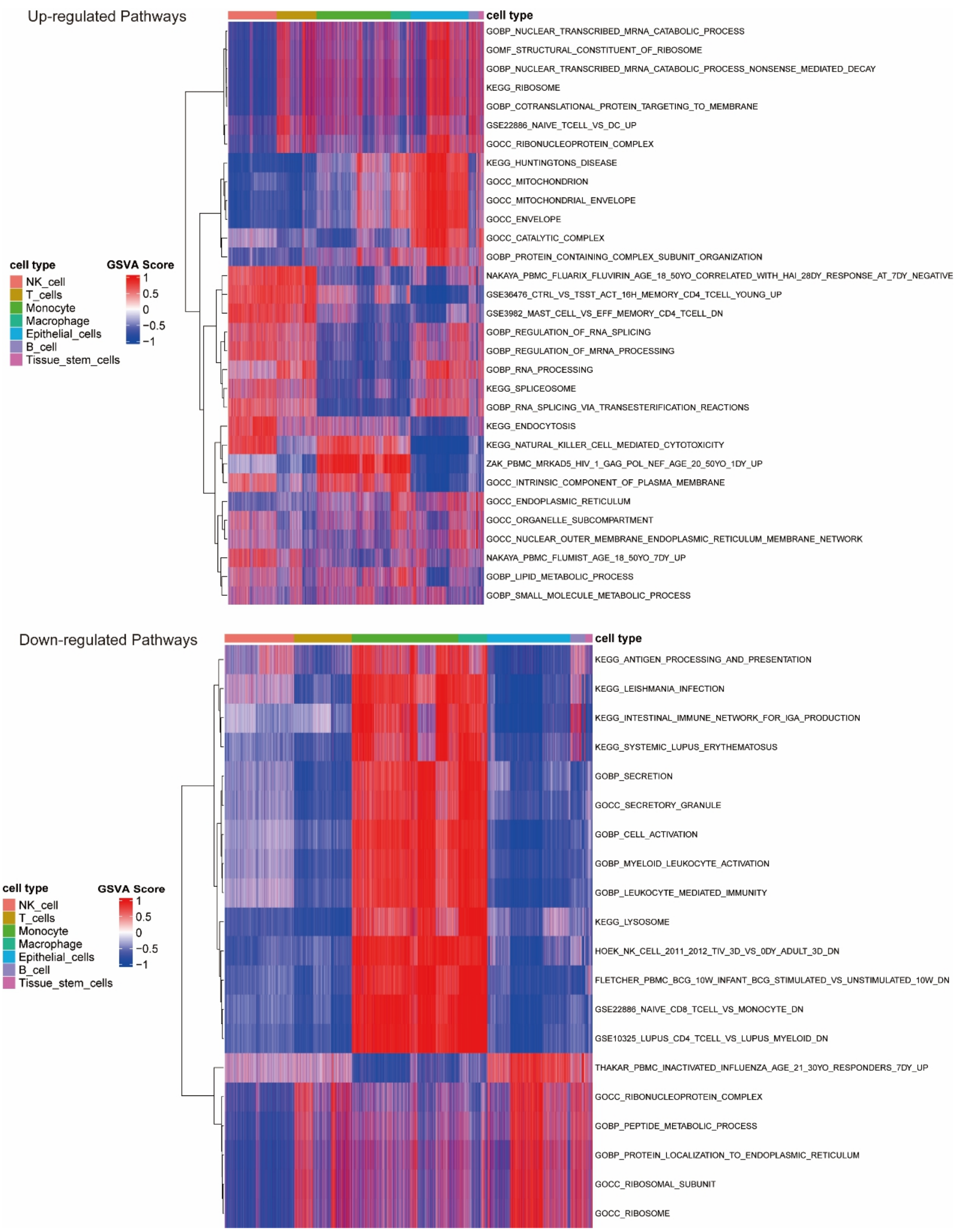

Figure S2. GSVA scores for up-regulated and down-regulated pathways in each cell type identified from scRNA-seq data. Abbreviations: GSVA, gene set variation analysis; scRNA-seq, single-cell RNA-seq

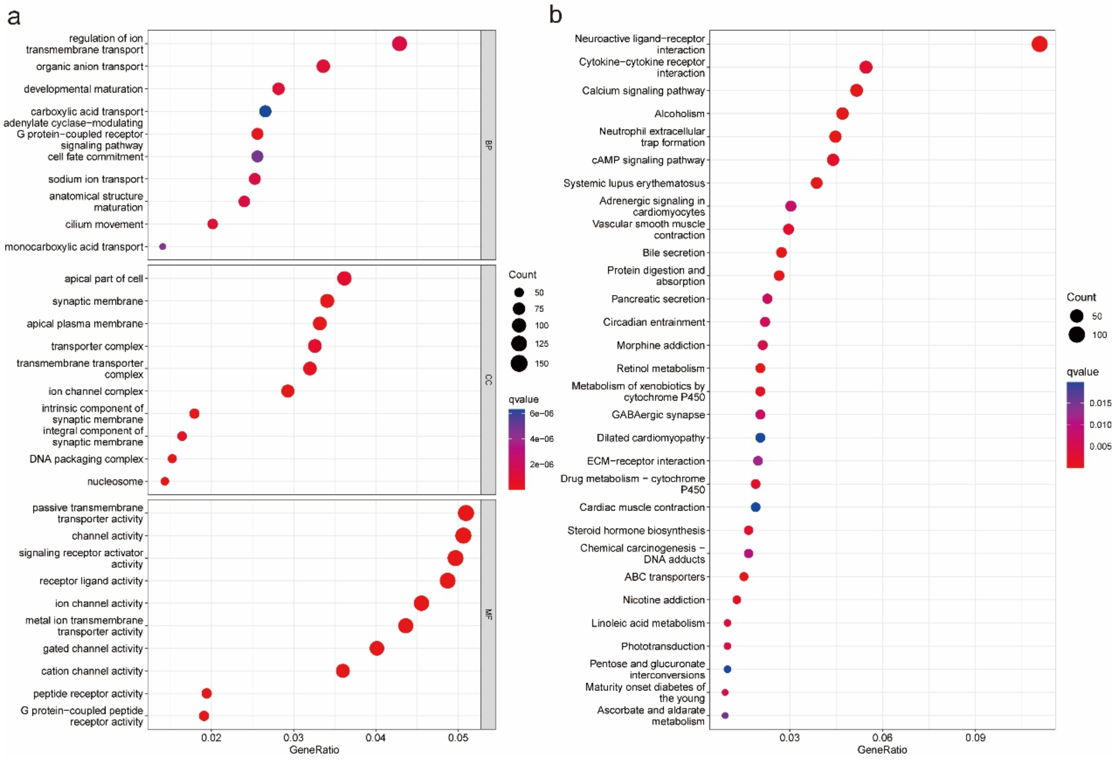

Figure S3. (a) A bubble plot to visualize the most significantly enriched pathways of DEGs through GO enrichment analysis. (b) A bubble plot to visualize the most significantly enriched pathways of DEGs through KEGG enrichment analysis. Abbreviations: DEGs, differential expression genes; GO, Gene Ontology; KEGG, Kyoto Encyclopedia of Genes and Genomes

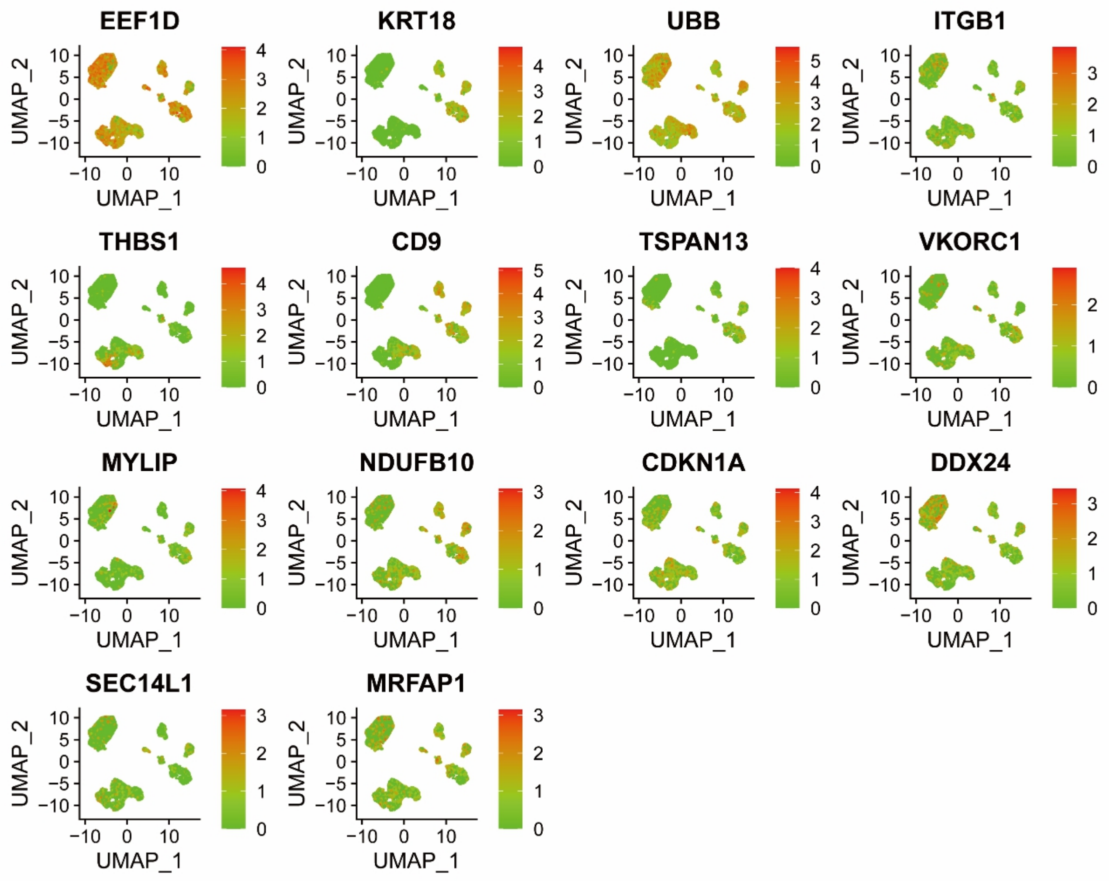

Figure S4. Heatmap for expression of constructed prognostic model genes in each cluster identified from scRNA-seq data. Abbreviations: scRNA-seq, single-cell RNA-seq

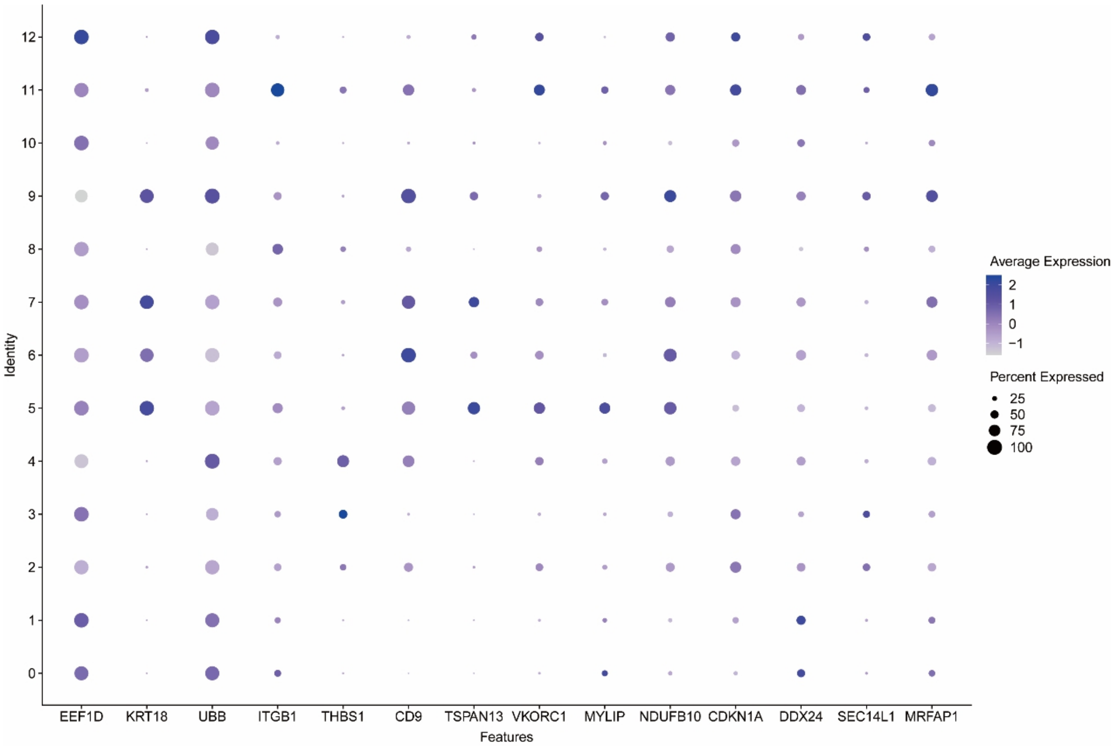

Figure S5. Bubble plot for expression of constructed prognostic model genes in each cluster identified from scRNA-seq data. Abbreviations: scRNA-seq, single-cell RNA-seq

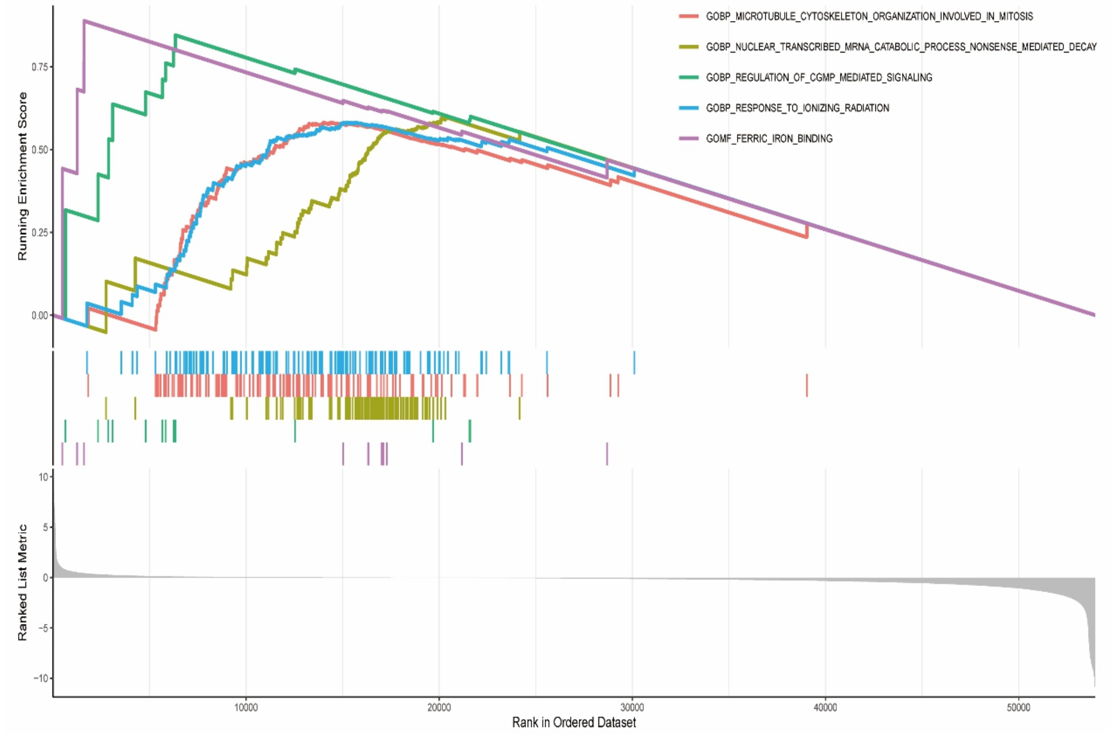

Figure S6. Up-regulated GO pathways for high-risk group via GSEA. Abbreviations: GO, Gene Ontology; GSEA, gene set enrichment analysis

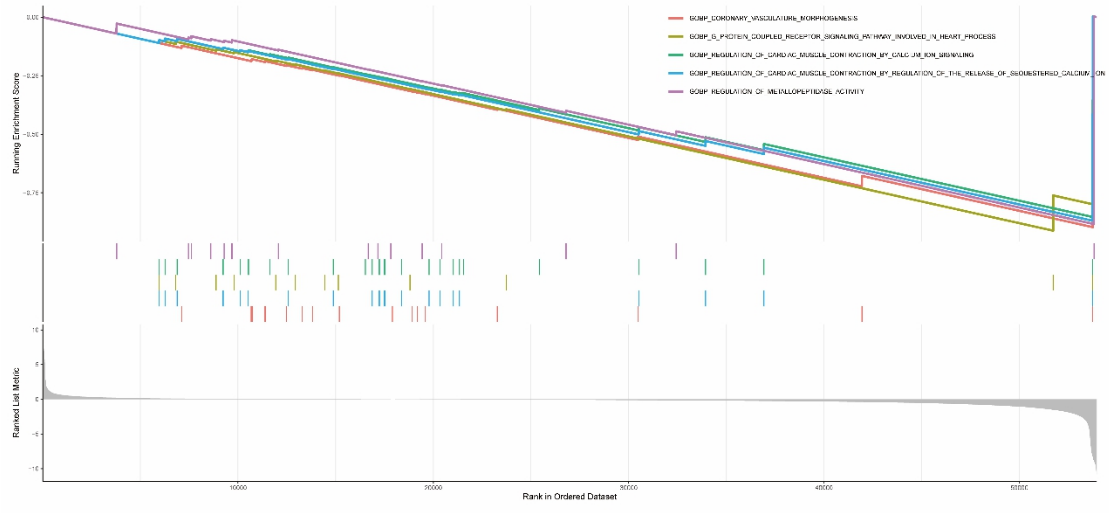

Figure S7. Up-regulated GO pathways for low-risk group via GSEA. Abbreviations: GO, Gene Ontology; GSEA, gene set enrichment analysis

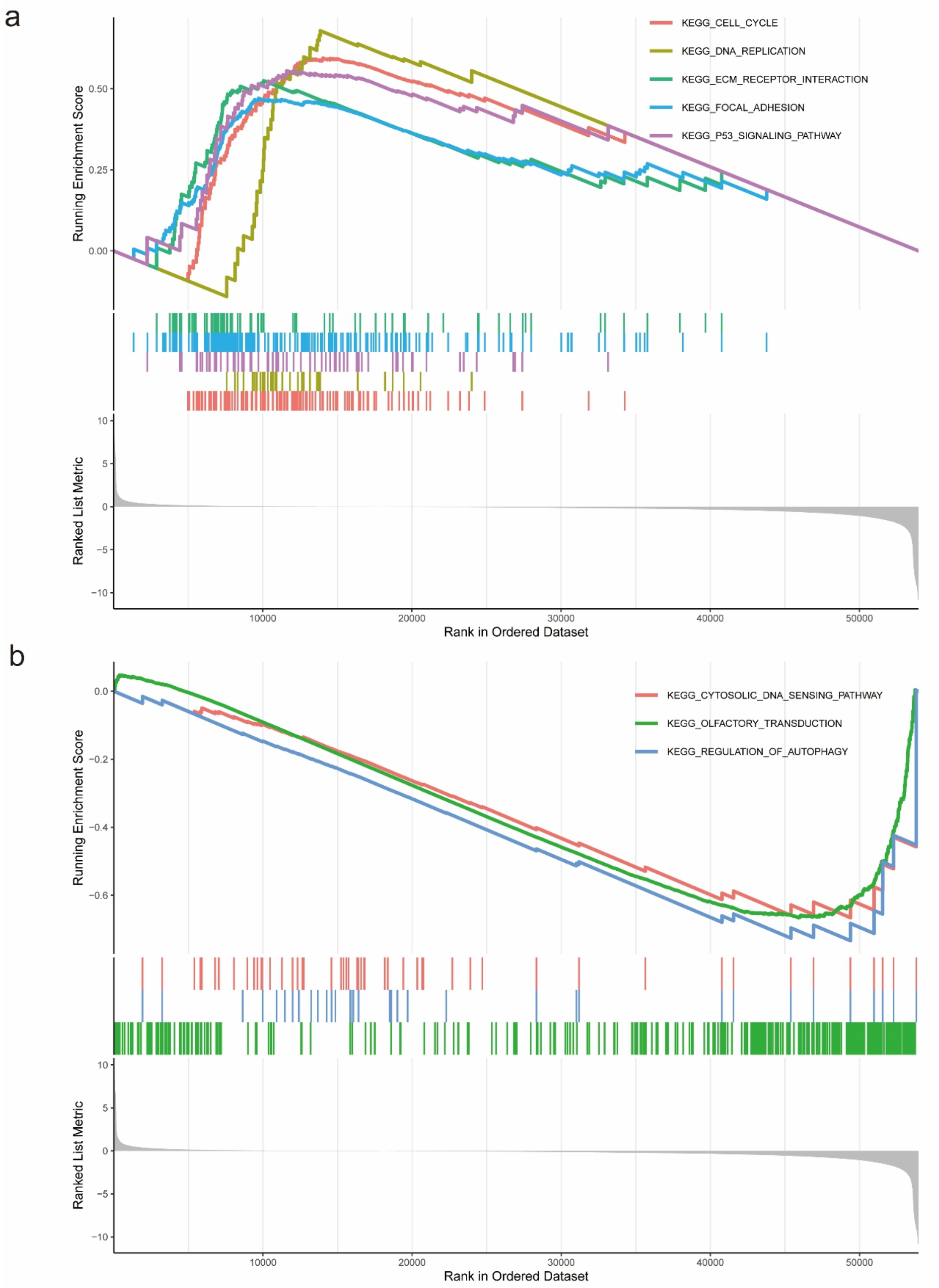

Figure S8. (a) Up-regulated KEGG pathways for high-risk group via GSEA. (b) Up-regulated KEGG pathways for low-risk group via GSEA. Abbreviations: KEGG, Kyoto Encyclopedia of Genes and Genomes; GSEA, gene set enrichment analysis

Table S1. Distribution of tumor cells and normal cells in each cluster

Cluster	C_T	C_N	P_T	P_N	Fold change (P_T/P_N)	Proportion test pvalue
C0	54	1814	2.9%	97.1%	0.03	< 2.2e-16
C1	548	1000	35.4%	64.6%	0.548	< 2.2e-16
C2	1010	374	73%	27%	2.701	< 2.2e-16
C3	184	822	18.3%	81.7%	0.224	< 2.2e-16
C4	574	196	74.5%	25.5%	2.929	< 2.2e-16
C5	689	3	99.6%	0.4%	229.667	< 2.2e-16
C6	10	548	1.8%	98.2%	0.018	< 2.2e-16
C7	283	253	52.8%	47.2%	1.119	0.21
C8	27	445	5.7%	94.3%	0.061	< 2.2e-16
C9	293	156	65.3%	34.7%	1.878	1.38e-10
C10	195	63	75.6%	24.4%	3.095	3.47e-16
C11	123	77	61.5%	38.5%	1.597	0.001
C12	78	63	55.3%	44.7%	1.238	0.238

* Abbreviations: C_T, count of tumor cells in each cluster; C_N, count of normal cells in each cluster; P_T, proportion of tumor cells in each cluster; P_N, the proportion of normal cells in each cluster.

Table S2. Drug Sensitivity Comparison Summary

Drug	Lower IC50	IC50 difference P-value	Correlation	Correlation P-value
Rapamycin	low-risk group	1.20e-06	0.18	9.90e-09
KIN001-102	low-risk group	2.00e-06	0.18	1.50e-08
KIN001-135	low-risk group	9.30e-06	0.17	5.60e-08
SB52334	low-risk group	2.70e-05	0.17	9.60e-08
GSK690693	low-risk group	4.00e-05	0.17	1.20e-07
ZSTK474	low-risk group	8.00e-05	0.15	1.90e-06
MP470	low-risk group	0.00012	0.17	9.80e-08
TL-2-105	low-risk group	0.00018	0.15	1.30e-06
FTI-277	high-risk group	< 2.2e-16	-0.33	< 2.2e-16
XAV939	high-risk group	2.90e-16	-0.32	< 2.2e-16
Cytarabine	high-risk group	7.60e-13	-0.29	< 2.2e-16
CCT018159	high-risk group	1.70e-12	-0.28	< 2.2e-16
Midostaurin	high-risk group	2.00e-12	-0.29	< 2.2e-16
JQ12	high-risk group	3.30e-12	-0.28	< 2.2e-16
AG-014699	high-risk group	3.60e-12	-0.26	6.60e-16
JNJ-26854165	high-risk group	5.00e-12	-0.26	< 2.2e-16
AMG-706	high-risk group	4.80e-11	-0.25	3.10e-15
Docetaxel	high-risk group	6.10e-11	-0.25	1.40e-15
BX-795	high-risk group	2.80e-10	-0.24	7.90e-14
Embelin	high-risk group	6.20e-10	-0.24	2.60e-14
Bleomycin (50	high-risk group	8.20e-10	-0.25	3.80e-15

uM)				
TW 37	high-risk group	1.20e-09	-0.25	3.60e-15
Bryostatin 1	high-risk group	1.20e-09	-0.23	1.40e-13
PFI-1	high-risk group	3.30e-09	-0.22	7.90e-12
MLN4924	high-risk group	4.30e-09	-0.22	2.60e-12
Vinblastine	high-risk group	5.30e-09	-0.22	3.60e-12
BEZ235	high-risk group	7.10e-09	-0.24	2.40e-14
CI-1040	high-risk group	7.60e-09	-0.22	3.10e-12
Pazopanib	high-risk group	8.10e-09	-0.22	4.40e-12
YK 4-279	high-risk group	1.20e-08	-0.22	2.30e-12
Temsirolimus	high-risk group	2.30e-08	-0.21	1.70e-11
Camptothecin	high-risk group	2.80e-08	-0.21	8.80e-11
LFM-A13	high-risk group	3.10e-08	-0.23	8.10e-13
Elesclomol	high-risk group	3.60e-08	-0.22	4.00e-12
BMS-509744	high-risk group	4.20e-08	-0.23	1.20e-12
RO-3306	high-risk group	5.70e-08	-0.21	2.50e-11
CEP-701	high-risk group	6.60e-08	-0.2	1.40e-10
SB 216763	high-risk group	6.90e-08	-0.22	3.20e-12
AZ628	high-risk group	7.60e-08	-0.2	3.10e-10
Bortezomib	high-risk group	9.20e-08	-0.22	1.30e-11
Thapsigargin	high-risk group	9.60e-08	-0.23	1.10e-12
NU-7441	high-risk group	1.10e-07	-0.21	6.40e-11
Bexarotene	high-risk group	1.50e-07	-0.23	1.30e-12
Trametinib	high-risk group	1.50e-07	-0.21	1.90e-11
PD-0332991	high-risk group	2.10e-07	-0.21	1.10e-10
PD-0325901	high-risk group	2.50e-07	-0.22	6.20e-12
JNK Inhibitor VIII	high-risk group	3.10e-07	-0.19	1.30e-09
Talazoparib	high-risk group	7.30e-07	-0.22	3.30e-12
VX-11e	high-risk group	1.10e-06	-0.18	2.90e-08
Bleomycin	high-risk group	1.20e-06	-0.23	1.10e-12
A-770041	high-risk group	1.40e-06	-0.2	3.20e-10
PF-562271	high-risk group	2.00e-06	-0.18	1.10e-08
HG-6-64-1	high-risk group	4.60e-06	-0.17	8.90e-08
681640	high-risk group	5.00e-06	-0.16	5.00e-07
(5Z)-7-Oxozeaenol	high-risk group	5.40e-06	-0.16	1.00e-06
Cisplatin	high-risk group	5.90e-06	-0.16	4.50e-07
AUY922	high-risk group	6.20e-06	-0.19	6.40e-09
HG-5-113-01	high-risk group	6.30e-06	-0.18	1.40e-08
17-AAG	high-risk group	6.80e-06	-0.2	5.10e-10
XMD8-92	high-risk group	1.20e-05	-0.15	1.50e-06
WH-4-023	high-risk group	1.30e-05	-0.17	9.80e-08
piperlongumine	high-risk group	1.30e-05	-0.17	1.50e-07
Foretinib	high-risk group	1.30e-05	-0.16	7.00e-07
Methotrexate	high-risk group	1.50e-05	-0.18	8.30e-09

Dasatinib	high-risk group	1.50e-05	-0.17	1.90e-07
Tamoxifen	high-risk group	1.80e-05	-0.16	5.60e-07
Saracatinib	high-risk group	1.90e-05	-0.18	7.70e-09
Tivozanib	high-risk group	2.20e-05	-0.15	4.20e-06
Dabrafenib	high-risk group	2.30e-05	-0.16	9.40e-07
AS601245	high-risk group	3.10e-05	-0.19	1.50e-09
Epothilone B	high-risk group	3.20e-05	-0.19	3.90e-09
Temozolomide	high-risk group	3.40e-05	-0.16	7.20e-07
Paclitaxel	high-risk group	5.00e-05	-0.15	5.80e-06
AZD7762	high-risk group	6.60e-05	-0.15	2.60e-06
WZ-1-84	high-risk group	8.40e-05	-0.13	7.00e-05
Axitinib	high-risk group	0.00013	-0.14	1.30e-05
TAE684	high-risk group	0.00014	-0.16	7.70e-07
BIRB 0796	high-risk group	0.00014	-0.13	4.30e-05
JNK-9L	high-risk group	0.00015	-0.14	8.80e-06
OSU-03012	high-risk group	0.0002	-0.15	3.10e-06
CGP-60474	high-risk group	0.00021	-0.16	7.80e-07
SN-38	high-risk group	0.00022	-0.14	8.20e-06
Nilotinib	high-risk group	0.00025	-0.13	7.20e-05
GSK-650394	high-risk group	0.00031	-0.15	2.20e-06
Lenalidomide	high-risk group	0.00031	-0.14	9.50e-06
CCT007093	high-risk group	0.00035	-0.13	5.00e-05
Sorafenib	high-risk group	0.00036	-0.12	0.00031
Pyrimethamine	high-risk group	0.00041	-0.14	2.40e-05
Etoposide	high-risk group	0.00042	-0.15	5.90e-06
AKT inhibitor VIII	high-risk group	0.00042	-0.13	2.80e-05
PD-173074	high-risk group	0.00042	-0.13	6.80e-05
CGP-082996	high-risk group	0.00043	-0.16	1.10e-06
Obatoclax Mesylate	high-risk group	0.00046	-0.13	7.10e-05
Gemcitabine	high-risk group	0.00047	-0.17	1.00e-07
GSK1904529A	high-risk group	0.00053	-0.15	5.20e-06
rTRAIL	high-risk group	0.0006	-0.16	8.10e-07
Doxorubicin	high-risk group	0.00068	-0.16	6.20e-07
QL-VIII-58	high-risk group	0.00068	-0.14	1.90e-05
Vinorelbine	high-risk group	0.00078	-0.13	5.40e-05
Veliparib	high-risk group	0.00085	-0.13	7.00e-05
HG-5-88-01	high-risk group	0.00096	-0.13	2.60e-05
Bosutinib	high-risk group	0.00096	-0.12	0.00024
